# Comparing Characteristics and Treatment of Brain Vascular Malformations in Children and Adults with HHT

**DOI:** 10.3390/jcm12072704

**Published:** 2023-04-04

**Authors:** Alexandra Kilian, Giuseppe A. Latino, Andrew J. White, Felix Ratjen, Jamie McDonald, Kevin J. Whitehead, James R. Gossage, Timo Krings, Michael T. Lawton, Helen Kim, Marie E. Faughnan, The Brain Vascular Malformation Consortium HHT Investigator Group

**Affiliations:** 1 Department of Paediatrics, The Hospital for Sick Children, Toronto, ON M5G 1X8, Canada; 2 Toronto HHT Centre, St. Michael’s Hospital, Li Ka Shing Knowledge Institute, Toronto, ON M5B 1W8, Canada; 3 Department of Pediatrics, North York General Hospital, University of Toronto, Toronto, ON M2K 1E1, Canada; 4 Department of Pediatrics, St Louis University, St. Louis, MO 63103, USA; 5 Division of Respiratory Medicine and Translational Medicine, The Hospital for Sick Children, Toronto, ON M5G 1X8, Canada; 6 Department of Pathology, University of Utah, Salt Lake City, UT 84132, USA; 7 Department of Medicine, Division of Cardiovascular Medicine, University of Utah, Salt Lake City, UT 84132, USA; 8 Department of Pediatrics, Division of Pediatric Cardiology, University of Utah, Salt Lake City, UT 84132, USA; 9 Department of Medicine, Augusta University, Augusta, GA 30912, USA; 10 Division of Neuroradiology, Toronto Western Hospital, University Health Network, Toronto, ON M5T 2S8, Canada; 11 Department of Neurosurgery, Barrow Neurological Institute, Phoenix, AZ 85013, USA; 12 Center for Cerebrovascular Research, Department of Anesthesia and Perioperative Care, University of California San Francisco, San Francisco, CA 94110, USA; 13 Institute for Human Genetics, University of California San Francisco, San Francisco, CA 94143, USA; 14 Department of Epidemiology and Biostatistics, University of California San Francisco, San Francisco, CA 94158, USA; 15 Division of Respirology, Department of Medicine, University of Toronto, Toronto, ON M5S 3H2, Canada

**Keywords:** hereditary hemorrhagic telangiectasia, pediatrics, brain vascular malformation, embolization, gamma knife, surgery, intracranial hemorrhage, screening

## Abstract

Hereditary hemorrhagic telangiectasia (HHT) is a rare autosomal dominant disease characterized by the development of vascular malformations (VMs) in organs such as the brain and lungs, as well as telangiectases on mucosal surfaces. Prophylactic treatment of organ VMs may prevent potential complications, such as hemorrhage. However, brain VM treatment—surgical resection, embolization, and/or radiosurgery—is not recommended for all patients due to the associated risks. Given the scarcity of data regarding HHT-related brain VM presentation and treatment trends in pediatric patients, we aim to describe the clinical presentations and the patterns of treatment of HHT-related brain VMs in a pediatric cohort, and compare pediatric trends to those of adults. Demographic and clinical data were analyzed in 114 pediatric patients with HHT-related brain VMs and compared with a cohort of 253 adult patients enrolled in the multicenter Brain Vascular Malformation Consortium HHT Project. Our data demonstrated that a higher proportion of pediatric patients with HHT-related brain VMs were symptomatic at presentation (*p* = 0.004). Moreover, a higher proportion of pediatric patients presented with intracranial hemorrhage (*p* < 0.001) and seizure (*p* = 0.002) compared to adult patients. Surgical resection was the most common brain VM treatment modality in both children and adults. We conclude that pediatric patients may be more likely to present with symptoms and complications from brain VMs, supporting the case for screening for brain VMs in children with HHT.

## 1. Introduction

Hereditary hemorrhagic telangiectasia (HHT) is a rare autosomal dominant disease with an estimated prevalence of 1 in 5000 [1,2,3]. It is mainly caused by mutations in three known genes; Endoglin (*ENG*), Activin A Receptor Like Type 1 (*ACVRL1*) and SMAD family member 4 (*SMAD4*), as well as in rare families, with mutations in Growth Differentiation Factor 2 (*GDF2*) or RAS P21 Protein Activator 1(*RASA1)*. In addition to telangiectases in the nasal mucosa and gastrointestinal (GI) tract, resulting in epistaxis and GI bleeding, patients can present with pulmonary arteriovenous malformations (AVMs) and brain, spine and liver vascular malformations (VMs) [4].

HHT-related brain VMs occur in approximately 10% of patients with HHT, based on a 2017 meta-analysis and systematic review [5]. Brain VMs are known to be more common in patients with an *ENG* mutation. Patients with HHT often have multiple brain VMs [6,7] and multiplicity has been found to be predictive of HHT diagnosis [5,8,9]. The complications of brain VMs, including rupture with intracranial hemorrhage (ICH), are associated with significant morbidity and a high risk of mortality. In patients with non-HHT related brain VMs, there is evidence that children may be more likely to present with ICH than in adults [10], despite a lower frequency of angio-architectural features typically associated with elevated risk of hemorrhage, such as aneurysms and venous outflow stenoses [11]. There is no published direct comparison of hemorrhage rates between adults and children in the HHT population.

The risks of hemorrhage, neurological deficits, and infection accompany all treatment options for brain VMs, namely surgery, stereotactic radiosurgery, and embolization [12,13,14,15]. As such, the decision to treat, and with which modality, is individualized for each patient [14]. The risk of treatment complications is weighed against the risks of spontaneous rupture and ICH of an untreated lesion. Given that identification of a brain VM via screening may not always result in preventative treatment, brain VM screening has been a subject of international controversy. There was insufficient expert agreement to recommend routine screening for brain VMs at the development of the 2009 International Guidelines [14], though brain VM screening for children with HHT was recommended in the 2020 HHT Guidelines [16]. Current practice trends suggest that screening for brain VMs is the North American standard of practice [17], while screening is uncommon in Europe [18], and remains controversial internationally.

There is a relative scarcity of evidence describing the clinical manifestations and treatment trends for HHT-related brain VMs, particularly in children. In response to this, and the international controversy surrounding the role of brain VM screening, we report and compare data on the clinical manifestations and treatment trends for HHT-related brain VMs from a large pediatric and adult cohort of patients.

## 2. Materials and Methods

### 2.1. Cohort

HHT patients with a brain VM from the Brain Vascular Malformation Consortium (BVMC) HHT Project were included in the study cohort. Demographic and clinical data were collected from 114 pediatric patients (age at brain VM diagnosis < 18 years) and 253 adult patients (age at brain VM diagnosis ≥ 18 years) enrolled in the BVMC HHT Project. All patients had a diagnosis of HHT and all patients had one or more brain VMs. The BVMC HHT cohort aims to recruit a cohort in which 25% of patients have a brain VM. Other clinical characteristics of the cohort are similar to other cohorts of HHT patients [19,20]. For the purpose of our study, arteriovenous fistulae (AVF), nidus type AVM, and capillary VM qualified as HHT related brain VMs [21].

The BVMC HHT Project includes 1679 HHT patients with a definite clinical or genetic diagnosis of HHT, enrolled at multiple recruiting centers in the US, Canada, and the Netherlands between 2010 and 2019. Cohort recruitment has been previously described [22,23]. Informed written consent was obtained from all patients (or guardians) in order to be included in all BVMC related projects. The study protocol was approved by the institutional review board at each recruiting center. Patients were screened for organ VMs and other clinical features according to standard clinical practice and International HHT Guidelines [14]. This typically included: comprehensive history, physical exam, and routine investigations; pulmonary AVM screening with contrast echocardiography or positional oximetry (protocols vary by center); brain VM screening by magnetic resonance imaging (MRI); clinical screening for liver VMs (chronic right upper quadrant pain, portal hypertension, high-output heart failure, liver bruit on examination); clinical screening for recurrent spontaneous epistaxis (>1 episode per month for >1 year); and screening for HHT-related GI-bleeding (history of anemia, iron deficiency, known GI telangiectases on endoscopy, melena, rectal bleeding). If screening was positive for pulmonary AVM or brain VM, patients underwent confirmatory diagnostic imaging and treatment where appropriate. If clinical assessment was suggestive of symptomatic liver VM, diagnostic imaging was recommended, along with therapy where appropriate. If initial clinical assessment was suggestive of GI bleeding, diagnostic endoscopy was recommended and endoscopic, medical, and supportive therapies were undertaken on a case-by-case basis. Decisions regarding brain VM treatment were made on a case-by-case basis by the clinical multidisciplinary team of the HHT treatment center. Data was collected both retrospectively at the time of enrolment and prospectively, during the period of data collection.

### 2.2. Analysis

We calculated the proportions of pediatric and adult patients with HHT-related brain VMs who presented with each clinical characteristic (asymptomatic, ICH, stroke, focal deficits, seizure, headache) and the proportion of patients in each group who underwent treatment, and by which modality. The two-sample z-test was used to compare proportions of various variables (clinical characteristics, treatment modalities) between the two cohorts (pediatric and adult patients). All *p*-values calculated were two-sided and significance was defined at *p* < 0.05.

## 3. Results

### 3.1. Demographics

54.4% of pediatric patients were female, compared with 63.6% of adult patients. This difference was not statistically significant. In the pediatric cohort, the average age at diagnosis of brain VM was 9.2 years (range: birth-17 years). In the adult cohort, the average age at diagnosis was 41.1 years (range: 18–77 years).

### 3.2. Clinical Presentation of HHT-Related Brain VMs

A significantly smaller proportion of pediatric patients with HHT and brain VMs were diagnosed by screening (*p* = 0.034); concordantly, a significantly higher proportion of pediatric patients were symptomatic at presentation (*p* = 0.004). The most common symptom at presentation was headaches for both pediatric and adult patients. Almost one-quarter of our pediatric cohort (23.7%) had ICH at presentation. A higher proportion of pediatric patients presented with intracranial hemorrhage (*p* < 0.001), compared to their adult counterparts (9.9%). Pediatric patients also experienced seizures at presentation in a higher proportion (18.3%) than adults (7.5%) (*p* = 0.002). Of note, patients may present with multiple symptoms, and in our data focal neurologic deficits refer to those unrelated to stroke or hemorrhage.

Demographic and clinical characteristics of pediatric and adult patients with HHT-related brain VMs in our cohort are summarized in Table 1.

### 3.3. Treatment of HHT Related Brain VMs

Table 2 describes treatment modalities and complications in both patient groups.

Sixty percent (60%) of pediatric patients with brain VMs underwent treatment compared to 51% of adults. This difference was not statistically significant. Surgical resection was the most common brain VM treatment modality in pediatric and adult patients with HHT.

Ten children and 31 adults underwent treatment using multiple modalities. Seven children and 18 adults underwent surgery and embolization. Two pediatric patients underwent gamma knife radiosurgery and embolization, compared to seven adult patients. Five adult patients underwent gamma knife radiosurgery and surgery, compared to one pediatric patient. One adult patient was treated with all three modalities; no pediatric patients were treated with the same.

There was no difference in the cumulative incidence of post-treatment hemorrhage (Table 2), nor when the incidence of post-treatment hemorrhage was analysed by intervention type (Table 3). The timing of post-treatment hemorrhage by intervention is captured in Table 4. Of note, for Table 3 and Table 4, hemorrhage is related to the treatment modality preceding ICH for patients who underwent multiple modalities, hence representing cumulative proportions.

### 3.4. Treatment for Brain VMs Complicated by ICH

Table 5 describes and compares treatment modalities in pediatric and adult patients with brain VMs complicated by pre-intervention ICH.

A significantly greater proportion of adult (23/27, 85%, *p* < 0.001) and pediatric patients (22/25, 88%, *p* = 0.002) with brain VMs complicated by ICH at initial presentation underwent treatment. Comparatively, brain VMs were treated in 46/87 (52.9%) of pediatric patients without initial ICH and 107/228 (46.9%) of adults without initial ICH. Surgery was the most common treatment modality in both pediatric (17/27, 63%) and adult patients (15/25, 60%) with ICH at initial presentation. Radiosurgery and multiple modalities were uncommon in both adult and pediatric patients with initial ICH. Overall, there was no statistically significant difference in the treatment trends of pediatric and adult patients presenting with ICH.

## 4. Discussion

Our data illustrates key differences between adult and pediatric patients with HHT-related brain VMs.

In our cohort, the majority of pediatric patients were symptomatic, compared to a minority of adult patients. A systematic review and meta-analysis found that 50% of HHT patients with brain VMs have symptoms related to brain VM, including headache, seizure, and/or focal neurological deficits [5]. Previous studies suggest that between 40% to 79% of patients with HHT and brain VMs are asymptomatic from the perspective of their brain VMs [8,24,25]. This range aligns with the percentage of patients that were asymptomatic within our adult and pediatric cohorts. As in previous cohorts, the most common symptom was headache [7,24,26]. The age-dependent nature of many HHT-related symptoms might explain, in part, why significantly higher proportion adults were asymptomatic at time of brain VM diagnosis and were diagnosed by screening. Mucocutaneous telangiectases and spontaneous recurrent epistaxis—two of the four Curacao criteria—are more prevalent with older age [27,28,29]. The absence or mild nature of these features in children can result in delayed diagnosis [4,14,30,31]. Moreover, the additional considerations associated with neuroimaging in children, including the need for sedation, may delay pediatric screening at the time of diagnosis [14,32]. Thus, a greater portion of adult patients may have been diagnosed with HHT following presentation with other features of HHT, and were subsequently screened for brain VMs. In contrast, for pediatric patients, the symptoms of brain VMs—headache, seizures, neurological deficits, among others—may have brought them to medical attention, ultimately prompting neuroimaging and the subsequent diagnosis of HHT, as suggested previously [25]. It is also important to acknowledge here that neurological symptoms are not all likely to be related to brain VMs, as pulmonary AVMs can result in paradoxical emboli, brain abscess and ischemic stroke and, more broadly, headaches in children have multifactorial etiologies.

Our data demonstrate an increased prevalence of ICH at presentation in children compared to adults. Similarly, the data from pediatric patients with non-HHT-related brain VMs also show that pediatric patients are more likely to present with ICH, compared to adults [10,33,34]. This has been explained by the finding that brain VMs are less likely to be diagnosed incidentally in pediatric patients, and that the initial bleed brings these patients to medical attention [34]. This is reflected in our data, which demonstrate that pediatric patients are more likely to be symptomatic at presentation, rather than be diagnosed by screening. Fullerton et al., demonstrated that, while pediatric patients with non-HHT related brain VMs were more likely to initially present with intracranial bleeding, the risk of subsequent hemorrhage was not increased compared to adults [33]. Given the current guideline support screening for adults, partially based on risk of hemorrhage, this finding further supports screening at diagnosis in the pediatric population. Finally, in the non-HHT population, an age-dependent trend in the risk of hemorrhage has also been described [35,36].

For HHT-related brain VMs, the rate of rupture and ICH has been difficult to quantify. The first study of bleeding related to brain VMs in HHT by Willemse et al. suggested that the bleeding rate in HHT may be lower than in sporadic brain VMs [37]. Similarly, Maher et al., suggested that ICH risk in patients with HHT-related brain VMs is low, after reporting that only 7 of 321 patients presented with ICH over 20-year period [38]. The evidence regarding a lower rate of rupture for HHT-related brain VMs compared to sporadic VMs, as well as the results of the ARUBA trial—a prospective, randomized controlled trial, which showed superior outcomes with medical management compared to intervention for patients with unruptured AVMs [13]—have fueled the international controversy around screening. However, the ARUBA trial specifically excluded pediatric patients. This and other identified methodological limitations call into question the applicability of its conclusions with regards to HHT-related brain VM management in pediatric patients [39,40].

There is a body of literature that suggests that the hemorrhage rate of HHT-related brain VMs approximates that of the non-HHT population [41,42]. In 2015, in a cohort of 153 patients, Kim et al. suggested that the rupture rate of HHT-related brain VMs approximates that of sporadic AVMs, particularly for previously ruptured AVMs. In this cohort, the rupture rate was 0.4% per year for unruptured VMs, and rose to 10% per year for previously ruptured lesions [41]. In another study of 44 patients, 10% of patients presented with ICH [6]. Finally, a systematic review concluded that approximately 20% of HHT patients with brain VMs will suffer ICH [5].

While the evidence regarding the hemorrhage risk for pediatric patients with HHT-related brain VMs arises from smaller cohorts, these cohorts do report a higher rate of ICH, compared to those reported in adult patients. ICH was the most common presentation of an HHT-related brain VM in a pediatric cohort of 61 patients [43]. In another pediatric cohort of 11 patients with HHT-related brain VMs, 55% patients (6/11) had ICH secondary to brain VMs [44]. Importantly, in this cohort, in 4/11 cases, ICH was a presenting feature of HHT [44]. In a cohort of 44 pediatric patients with HHT, seven had brain VM (15.9%). Two of these seven children had brain VM related complications; with one presenting with ICH and the other with seizures [45]. In a cohort of 171 pediatric and adult patients, 27% patients had a history of ICH, which approximates the rate in our pediatric cohort [19]. Morgan et al. reported a case series of nine pediatric patients with HHT-related brain VMs who suffered catastrophic consequences; five patients passed away and four patients were left with significant motor and cognitive impairment. The authors argue that screening may prevent such catastrophic sequelae [46]. Furthermore, a 2006 literature review concluded that ICH related to brain VMs was the leading cause of death in the pediatric HHT population, as reflected in the literature [47]. The higher rate of ICH in childhood may be related to the increased prevalence of (AVF), which are more commonly present in pediatric patients and carry a higher rate of hemorrhage than other VM subtypes [15,18,25,27]. Given that our pediatric cohort demonstrated increased prevalence of ICH at presentation, and a higher symptom burden, compared to adults, we emphasize that children may particularly benefit from screening to prevent the potential sequelae of brain VMs.

There are very few pediatric specific studies to guide treatment for pediatric patients with HHT-related brain VMs. Surgery was the most common modality in pediatric and adult patients in our cohort. It was also the most common modality in patients presenting with ICH in pediatric and adult patients; this is likely attributable to the fact that surgery allows for decompression and shunt placement when required [48]. The largest surgical cohort of patients with HHT-related brain VMs showed that surgical treatment was both safe and had favorable outcomes when compared to a cohort that did not undergo surgical treatment [26]. In the untreated cohort, functional status worsened at a rate of almost 1.5 times that of the surgically treated group [26]. Given that embolization is frequently used as an adjunct to surgery and radiosurgery (in order to decrease flow to the brain VM, reduce the size of the brain VM or decrease the risk of blood loss prior to surgery or radiosurgery [48]), it follows that embolization was commonly a component of multi-modal treatment plans in our series, but a rare standalone treatment. The evidence for embolization in pediatric patients with HHT remains limited. In a series of 31 pediatric patients with HHT-related brain VMs who underwent embolization, 60% of patients had symptomatic improvement or achieved occlusion, two patients (6.5%) had a persistent new neurological deficit, and two patients (6.5%) died as a result of the embolization [15]. In 19/31 of these patients, there was incomplete embolization. There was no subsequent rebleed or complications over the follow-up period, leading the authors to hypothesize that embolization could still be therapeutic [15]. Radiosurgery has been shown to be effective and safe in small cohorts of adult [49] and pediatric [50] patients with HHT, especially as brain VMs in HHT are often smaller than sporadic AVMs [9,25,49]. In a pediatric series of patients with non-HHT-related brain VMs, stereotactic radiosurgery was effective for smaller AVMs [51]. Given that radiosurgery is more likely chosen as the treatment modality in smaller brain VMs for which surgery or embolization is not feasible [52,53], future analyses comparing treatment trends would benefit from reporting VM size.

Our conclusions must be interpreted in the context of the limitations of our study. The targeted recruitment of patients with brain VMs may predispose to the recruitment of symptomatic patients or patients with more severe disease, which may have impacted the treatment trends we present. Patients with more severe or symptomatic disease may be more likely to be selected for therapeutic interventions, may be more likely to undergo multiple interventions, and/or experience treatment complications. However, given that this recruitment bias is assumed to affect the two populations being compared, it would follow that it should not significantly affect our ability to compare treatment trends between pediatric and adult patients with HHT. Additionally, the data collected in the BVMC HHT project is limited by its retrospective nature. Finally, our analysis did not include brain VM size, location, multiplicity, or subtype, which may impact choice of treatment intervention and thus treatment trends.

Despite these limitations, the large sample size and multi-center nature of our data supports the generalizability and validity of our results. Moreover, the characteristics described in our sample are similar to previously published pediatric and adult HHT populations [5]. The similarities between our sample and previously described clinical characteristics of pediatric and adult patients with non-HHT brain VMs suggest that our results may be generalizable to this population also, with appropriate caveats.

## 5. Conclusions

Our data, from a large cohort of pediatric and adult patients with HHT, suggests that pediatric patients with HHT present with symptoms and complications of undiagnosed brain VM at higher rates compared to adults with HHT; brain AVM rupture may be the first symptom of HHT in children and can be devastating. Surgical resection was the most common brain VM treatment modality in pediatric and adult patients with HHT. Our large data cohort supports the current recommendation for screening for brain VMs in pediatric patients and supports the case for systematic brain VM screening for adult patients with HHT.

## Figures and Tables

**Table 1:** Demographic and clinical characteristics of pediatric and adult HHlT patients with Brain VMs.

Characteristic (*n*, %)	Pediatric Patients *n* = 114	Adult Patients *n* = 253	*p*-Value
Female	62 (54.4%)	161 (63.6%)	0.076
Age at diagnosis (years)			-
Mean	9.2	41.1	
Standard deviation	±5.5	±14.6	
Median	10	40	
Range	Birth-17	18–77	
Interquartile Range	5–14	28–54	
Number of brain VMs (range)	1–4	1–10	-
>1 brain VM	15 (13.1%)	38 (15.0%)	0.638
Diagnosed by screening	63 (56.1%)	168 (66.4%)	0.034
Asymptomatic at presentation	54 (47.4%)	160 (63.2%)	0.004
ICH at presentation	27 (23.7%)	25 (9.9%)	<0.001
Ischemic stroke at presentation	14 (12.3%)	24 (9.5%)	0.417
Focal neurological deficit	10 (8.8%)	30 (11.9%)	0.379
Seizure	21 (18.4%)	19 (7.5%)	0.002
Headache	34 (29.8%)	53 (20.9%)	0.064

Hereditary Hemorrhagic Telangiectasia (HHT); Vascular Malformation (VM); Intracranial Hemorrhage (ICH).

**Table 2:** Treatment Modalities for Pediatric and Adult patients with HHT related brain VMs.

Treatment Modality (*n*, %)	Pediatric Patients *n* = 114	Adult Patients *n* = 253	*p*-Value
Treatment for brain VM	69 (60.5%)	128 (50.6%)	0.076
	*n* = 69	*n* = 128	
Surgery only	34 (49.3%)	43 (33.5%)	0.032
Embolization only	19 (27.5%)	16 (12.5%)	0.008
Radiosurgery only	6 (8.7%)	38 (29.7%)	<0.001
Multiple modalities	10 (14.58%)	31 (24.2%)	0.110
Surgery + Embolization	7 (10.1%)	18 (14.1%)	0.429
Surgery + Radiosurgery	1 (1.5%)	5 (3.9%)	0.246
Embolization + Radiosurgery	2 (3.0%)	7 (5.4%)	0.337
Embolization + Radiosurgery + Surgery	0 (0.0%)	1 (0.01%)	0.459
Post-treatment hemorrhage	3 (4.3%)	9 (6.9%)	0.453

Hereditary Hemorrhagic Telangiectasia (HHT); Vascular Malformation (VM). Of note, this table includes all patients who underwent treatment, including those treated prophylactically.

**Table 3:** Incidence of Post-treatment Hemorrhage by Intervention.

	Incidence of Post-Treatment Hemorrhage		
Intervention	Pediatric Patients	Adult Patients	*p*-Value
Surgery	0/42 (0.0%)	1/67 (1.4%)	0.423
Embolization	2/28 (7.1%)	4/42 (9.5%)	0.726
Radiosurgery	1/9 (11.1%)	4/51 (7.8%)	0.741

**Table 4:** Timing of Post-treatment Hemorrhage by Intervention.

	Timing of Post-Treatment Hemorrhage					
Intervention	Acute (<30 Days)		Remote (>30 Days)		Range	Missing Data
	Pediatric	Adults	Pediatric	Adults		
Surgery	0/42 (0.0%)	0/67 (0.0%)	0/42 (0.0%)	1/67 (1.4%)	15 years	0
Embolization	0/28 (0.0%)	2/42 (4.8%)	1/28 (3.5%)	1/42 (2.3%)	1–118 days	2
Radiosurgery	0/9 (0.0%)	0/51 (0.0%)	1/9 (11.1%)	1/51 (1.9%)	1–2 years	3

**Table 5:** Treatment for brain VMs complicated by ICH at initial presentation.

	Initial ICH		*p*-Value
	Pediatric (27/114, 23.7%)	Adult Patients (25/253, 9.9%)	
	*n* = 27	*n* = 25	
Underwent treatment for brain VM	23 (85.1%)	22 (88.0%)	0.764
Surgery	17 (63.0%)	15 (60.0%)	0.825
Embolization	6 (22.2%)	5 20.0%)	0.888
Radiosurgery	0 (0%)	5 (20.0%)	0.423
Multiple modalities	0 (0%)	3 (12.0%)	0.118

Hereditary Hemorrhagic Telangiectasia (HHT); Vascular Malformation (VM); Intracranial Hemorrhage (ICH).

## Data Availability

The data presented in this study are available on request from the corresponding author. The data are not publicly available due to privacy restrictions.
